# The complete mitochondrial genome of *Sarcophaga caudagalli* (Diptera: Sarcophagidae)

**DOI:** 10.1080/23802359.2019.1644552

**Published:** 2019-07-29

**Authors:** Xiao Shen, Yanjie Shang, Lipin Ren, Jifeng Cai, Yadong Guo

**Affiliations:** Department of Forensic Science, School of Basic Medical Sciences, Central South University, Changsha, China

**Keywords:** Mitochondrial genome, *Sarcophaga caudagalli*, Sarcophagidae

## Abstract

*Sarcophaga caudagalli* belonging to the Sarcophagidae is significant for medical and veterinary management. The complete mitochondrial genome (mitogenome) of S. *caudagalli* was first sequenced and annotated. The circular molecule was 15,029 bp in length, containing 37 genes (13 protein-coding genes (PCGs), two rRNA genes, 22 tRNA genes, and an A + T-rich region). Phylogenetic analysis showed that the clade of *S. caudagalli* was clustered separately.

Among the current number of species of flesh flies, *S. caudagalli* Bottcher, 1912 is mainly distributed in Asia, such as China, Japan, India, and Thailand, which is an important element of the carrion insect community (Lshijima 1967). The mitogenome has been widely used for phylogeny studies and molecular diagnostics (Shang et al. 2019). In this study, the complete mitogenome of *S. caudagalli* was 15,029 bp in length (Genbank No. MK820721), containing 37 genes (13 PCGs, two rRNA genes, 22 tRNA genes, and an A + T-rich region). Additionally, it exhibited characteristics of A (39.3%), G (9.6%), C (14.7%), and T (36.4%).

Adult specimens of *S. caudagalli* were captured in December 2018 in Jinghong, Yunnan province, China (21°27′N; 100°25′E). All specimens were frozen to death and stored under −80 °C subsequently. The specimens were identified according to the keys of morphological characteristics (Xu and Zhao 1996). The voucher specimens were assigned a unique field code (CSU19040903) and deposited in the Guo’s Lab (Changsha, Hunan, China). Genomic DNA was extracted using the QIANamp Micro DNA Kit, and then the complete mitogenome was sequenced on an Illumina HiSeq 2500 Platform.

Phylogenetic analysis was performed using Neighbour-joining method, and constructed with 13 concatenated PCGs, representing *S. caudagalli* and 11 other flesh flies. *Calliphora pinguis* and *Chrysomya vomitoria* belonging to Calliphoridae were used as an outgroup (Figure 1). The results showed that the clade of *S. caudagalli* was clustered separately, as well as *R. pernix*. The complete mitogenome of *S. caudagalli* provided important information for exploring phylogenetic analysis and population genetics.

**Figure 1.**
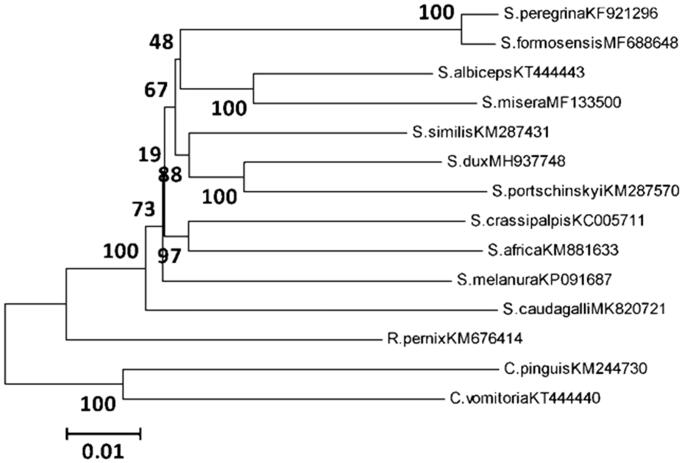
Phylogenetic analysis was performed using Neighbour-joining method, representing *S. caudagalli* and 11 other flesh flies. *Calliphora pinguis* and *C. vomitoria* were used as an outgroup.
